# Protective Role of Betulinic Acid against Cisplatin-Induced Nephrotoxicity and Its Antibacterial Potential toward Uropathogenic Bacteria

**DOI:** 10.3390/ph16081180

**Published:** 2023-08-18

**Authors:** Fatemah A. Alherz, Engy Elekhnawy, Hend Mostafa Selim, Thanaa A. El-Masry, Aya H. El-Kadem, Ismail A. Hussein, Walaa A. Negm

**Affiliations:** 1 Department of Pharmaceutical Sciences, College of Pharmacy, Princess Nourah Bint Abdulrahman University, P.O. Box 84428, Riyadh 11671, Saudi Arabia; faalherz@pnu.edu.sa; 2 Pharmaceutical Microbiology Department, Faculty of Pharmacy, Tanta University, Tanta 31527, Egypt; 3 Biochemistry Department, Faculty of Pharmacy, Tanta University, Tanta 31527, Egypt; hend.m.selim@pharm.tanta.edu.eg; 4 Department of Pharmacology and Toxicology, Faculty of Pharmacy, Tanta University, Tanta 31527, Egypt; thanaa.elmasri@pharm.tanta.edu.eg (T.A.E.-M.); aya.elkadeem@pharm.tanta.edu.eg (A.H.E.-K.); 5 Department of Pharmacognosy and Medicinal Plants, Faculty of Pharmacy (Boys), Al-Azhar University, Cairo 11884, Egypt; ismaila.hussein@azhar.edu.eg; 6 Department of Pharmacognosy, Faculty of Pharmacy, Tanta University, Tanta 31527, Egypt

**Keywords:** acute kidney injury, autophagy, oxidative stress, renoprotective, *Silene succulenta*, superoxide dismutase

## Abstract

Acute kidney injury (AKI) is one of the major side effects of cisplatin, a remarkable anticancer agent. Therefore, there is a growing need to find an agent that could mitigate cisplatin-induced nephrotoxicity. Betulinic acid (BA) is a natural compound isolated from *Silene succulenta* Forssk for the first time, with miraculous biological activities and no reports of its effect on the nephrotoxicity induced by cisplatin. Mice received BA orally with doses of 30 and 50 mg/kg before the intraperitoneal injection of cisplatin. Betulinic acid was found to decrease serum levels of creatinine and tissue levels of NGAL and kidney injury molecule (KIM-1) and improve the histological changes in the kidney. In addition, BA decreased the oxidative stress marker malondialdehyde (MDA), increased superoxide dismutase (SOD) antioxidative activity and suppressed the intensity of IL-1B and NFкB immuno-staining. Interestingly, betulinic acid enhanced autophagy by increasing beclin 1, ATG5, and LC3II and decreasing p62 expressions. Thus, our findings suggest betulinic acid as a potential agent that may protect from acute kidney injury by targeting inflammation, oxidative stress, and autophagy processes. Novel drugs are needed to combat the spreading of multidrug resistance between pathogenic bacteria, especially uropathogenic isolates. So, we elucidated the antibacterial properties of BA on *Pseudomonas aeruginosa*, *Escherichia coli*, *Proteus mirabilis*, and *Klebsiella pneumoniae*. Betulinic acid had minimum inhibitory concentration values (128 to 512 µg/mL). In addition, it adversely affected the membrane integrity of the tested isolates. Accordingly, betulinic acid should be clinically investigated in the future for urinary tract diseases.

## 1. Introduction

Acute kidney injury (AKI) is a significant issue for health systems, directly related to short and long-term morbidity and mortality. AKI is defined as a sudden deterioration of kidney function, which involves damage to the kidney structure and loss of function. It may occur due to several etiologies, such as renal ischemia, sepsis, infectious diseases, and nephrotoxic drugs, including cisplatin [1,2,3].

Cisplatin is one of the most common drugs used worldwide in the treatment of breast, lung, and bladder cancers. However, its clinical use is limited due to severe side effects, including nephrotoxicity and AKI [4]. Hence, mitigation of cisplatin-induced nephrotoxicity is a critical issue. The underlying pathophysiologic mechanisms of cisplatin-induced AKI should be investigated to provide new options for ameliorating nephrotoxicity induced by cisplatin [5]. It has previously been investigated that necrosis, oxidative stress, inflammation, and apoptosis might play critical roles in cisplatin-induced nephrotoxicity [6].

Autophagy is a conserved multistep pathway that degrades and recycles damaged organelles and macromolecules. It is regulated under stress conditions, including cell injury and oxidative stress, that are involved in the pathogenesis of AKI. Pyroptosis is a pathway activated as a result of oxidative stress and inflammation and was proven to modulate AKI [7]. Thus, modulation of autophagy may provide an alternative option for attenuating cisplatin-induced nephrotoxicity.

Bacterial infections in the urinary tract are common in a large part of communities worldwide. Among the most common bacterial species that cause urinary tract infections (UTI) are *Pseudomonas aeruginosa*, *Escherichia coli*, *Proteus mirabilis*, and *Klebsiella pneumoniae* [8]. Like most pathogenic bacteria, UTI-causing bacteria are acquiring resistance to multiple antibiotics, predisposing a major problem in treating such infections. This is in addition to the complications that could result from failure to treat such infections as sepsis and high mortality rate [9].

Natural sources like plants are rich in various pharmacologically active compounds that could be used as therapeutic drugs for various pathological disorders [10]. Betulinic acid is a pentacyclic Lupane-type triterpene found primarily in some medicinal herbs and plants, particularly in white birch bark. It shows a vast array of biological activities regarding fighting oxidative stress, inflammation, and tumor progression [11].

BA has recently shown a protective effect in kidney injury conditions by inhibiting inflammation [12]. However, the BA effect on the acute kidney injury induced by cisplatin and its possible modulation of oxidative stress, apoptosis, inflammation, and autophagy, in addition to its impact on renal bacterial infection, has not been investigated yet. So, our study is the first of its kind that aims to explore the possible beneficial effect of BA on cisplatin-induced renal injury-mediated pyroptosis in an albino mice animal model, in addition to its potential to act as a fighter against renal-bacterial infection.

## 2. Results

### 2.1. Phytochemical Study

The compound was obtained as a colorless amorphous powder. The HR-ESI-MS of the compound showed a peak at *m*/*z* 455.35823 [M-H]^−^, indicating that the molecular formula is C_30_H_48_O_3_ (Figure S1).

The ^13^C-NMR spectra revealed 30 carbon signals which were shown by the DEPT experiment to be six methyl groups, five quaternary carbons, one carboxylic acid, and two olefinic carbons suggesting that the compound is a triterpenic acid having five rings. The ^1^H-NMR spectrum presented the signals for five tertiary methyl groups at δ 1.23, 1.85, 1.02, 0.84, and 1.81, one isopropenyl moiety at δ 1.77, 4.96 and 4.78, indicating a Lupane-type skeleton (Table 1).

The compound was determined as 3β-hydroxy-lup-20(29)-en-28-oic acid, betulinic acid by directly comparing its spectral data with literature.

**Figure 1:**
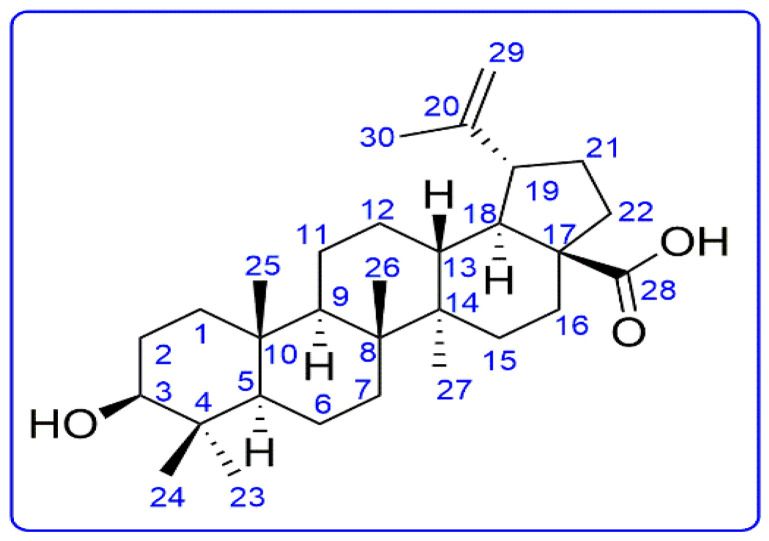
Chemical structure of the BA.

From the data mentioned above and the literature [13], the compound was elucidated as betulinic acid with molecular formula C_30_H_48_O_3_ (Figure 1).

### 2.2. Antibacterial Action

Betulinic acid demonstrated antibacterial action toward the uropathogenic bacterial isolates by agar well diffusion assay as there were inhibition zones around the well containing the betulinic acid. The values of the minimum inhibitory concentration (MIC, the lowest concentration that resulted in inhibition of bacterial growth) are shown in Table 2. The MIC value indicates that the agent has antibacterial activity on the test organism, and it reveals the lowest antibacterial concentration of this compound. The lower the MIC values, the higher the antibacterial activity of the test compound.

#### Influence of Betulinic Acid on the Membrane Integrity of the Tested Bacteria

**Figure 2:**
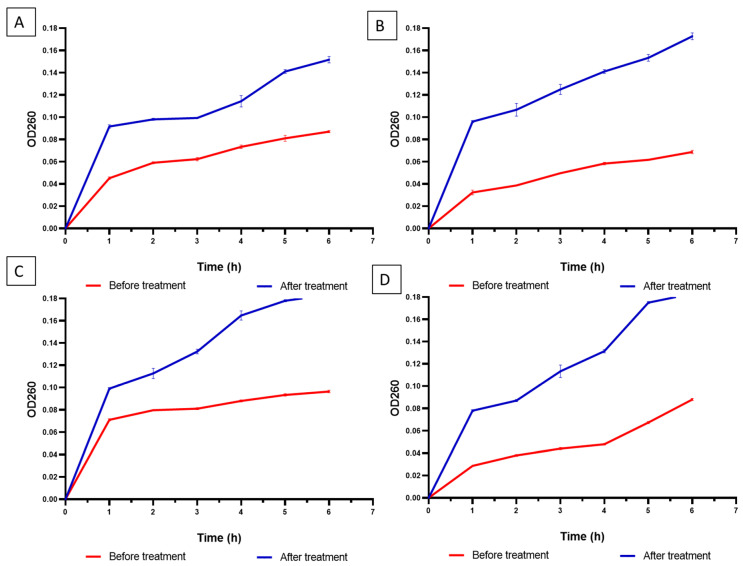
Impact of betulinic acid on the integrity of membranes (at 0.5 MIC values): (**A**) *P. aeruginosa*, (**B**) *E. coli*, (**C**) *P. mirabilis*, and (**D**) *K. pneumoniae* isolates. There was a significant decrease in the membrane integrity, as revealed by the significant increase (*p* < 0.05) in the 260 nm absorbing materials.

Betulinic acid exhibited a negative impact on the bacterial membrane integrity of the tested isolates, as shown in Figure 2.

### 2.3. In Vivo Activity

#### 2.3.1. Nephrotoxicity Serum Indices

**Figure 3:**
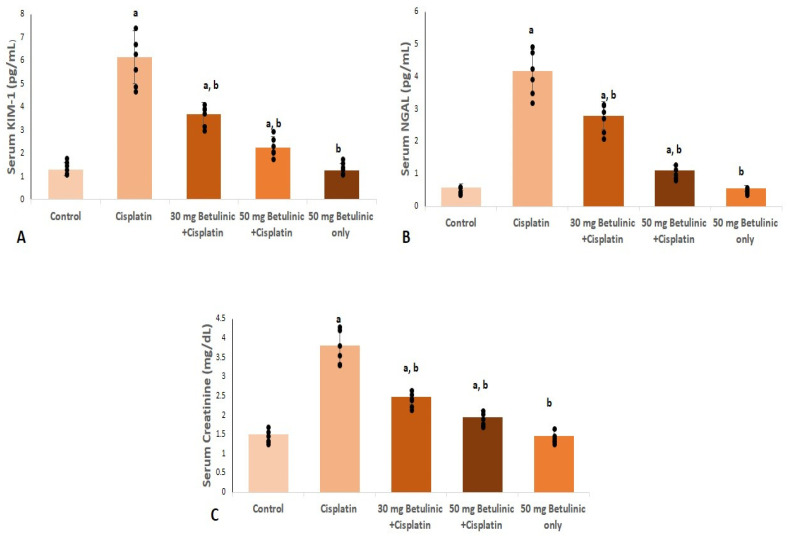
Effect of Betulinic acid pretreatment on (**A**) serum KIM-1 level, (**B**) serum NGAL level, and (**C**) serum creatinine. Cisplatin (25 mg/kg, i.p) was used for nephrotoxicity induction. Betulinic acid (30 mg/kg and 50 mg/kg orally) was given for 10 days. Data expressed as mean ± SD (*n* = 6/group), a means significant vs. control, b means significant vs. cisplatin group. *p* ˂ 0.05.

Their serum levels were measured for the reliability of both kidney injury molecule-1 (KIM-1) and Neutrophil gelatinase-associated lipocalin (NAGL) as markers for nephrotoxicity. Serum KIM-1 levels were significantly increased in the cisplatin group (4.7-fold increase) compared to the normal control group. On the contrary, mice treated with 30 and 50 mg/kg betulinic acid showed a remarkable decrease in KIM-1 (39.8% and 63.4%) relative to the cisplatin group (Figure 3A).

Likewise, cisplatin triggered a significant rise in serum NGAL levels (7.1-fold increase) relative to the control group, while betulinic acid treatment (30, 50 mg/kg) showed a marked decrease (32.7% and 73.5%, respectively) compared to cisplatin group (Figure 3B).

Cisplatin injection significantly increased the serum levels of creatinine (2.53-fold increase) compared to the control. However, compared to the cisplatin group, 30 and 50 mg/kg of betulinic acid pretreatment induced a considerable reduction in serum creatinine levels (35% and 48.7%, respectively), as shown in Figure 3C.

#### 2.3.2. Renal Oxidative Stress Markers

As shown in Table 3, cisplatin markedly triggered oxidative stress, for it caused a profound increase in renal lipid peroxidation reflected in a remarked increase in malondialdehyde (MDA) level (1.93-fold increase) compared to the control. Moreover, cisplatin significantly suppressed renal superoxide dismutase (SOD) activity (51.64%). On the other hand, 50 mg/kg BA pretreatment showed an alleviation in the oxidative stress represented as a remarked decrease in MDA level (34.6%) and remarked improvement in SOD renal activity (1.63-fold increase), respectively.

#### 2.3.3. Autophagy Biomarkers

##### Beclin 1 and Autophagy related 5 (ATG5) Gene Expression

**Figure 4:**
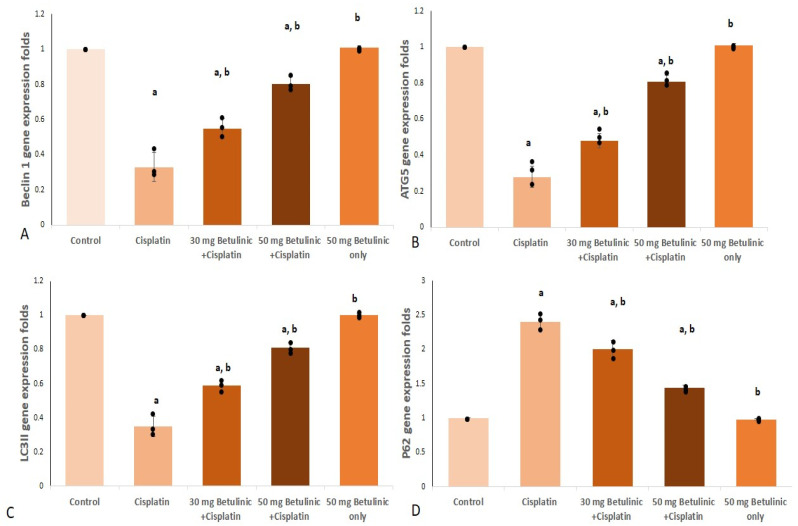
Effect of Betulinic acid pretreatment on (**A**) Beclin 1 gene expression, (**B**) ATG5 gene expression, (**C**) LC3II gene expression, (**D**) P62 gene expression. Cisplatin (25 mg/kg, i.p) was used for nephrotoxicity induction. Betulinic acid (30 mg/kg and 50 mg/kg, orally) was given for 10 days. Data expressed as mean ± SD (*n* = 3/group), a means significant vs. control, b means significant vs. cisplatin group. *p* ˂ 0.05.

The cisplatin group showed a remarkable decrease in Beclin 1 and ATG5 gene expression (67% and 72%, respectively) compared to the control. However, 30 and 50 mg/kg of BA pretreatment exhibited a significant increase in Beclin 1 (1.67- and 2.43-fold increase, respectively) and ATG5 (1.71- and 2.9-fold increase, respectively) gene expressions, compared to the cisplatin group as shown in Figure 4.

##### LC3 II and p62

Regarding LC3II, the positive control (cisplatin) remarkably suppressed (0.65% decrease) its gene expression relative to normal values. However, pretreatment with 30 and 50 mg BA significantly raised LC3II gene expression folds (1.66- and 2.3-fold increase, respectively, compared with the cisplatin groups) (Figure 4C).

Upon injecting cisplatin, a significant increase in p62 expression was observed (2.4-fold increase) compared to the control. On the other hand, 30 and 50 mg/kg of BA pretreatment partially corrected the cisplatin effect and significantly decreased p62 gene expression (16.6% and 40%, respectively) compared to the cisplatin group (Figure 4D).

#### 2.3.4. Beclin 1, ATG5, LC3 II and p62 Protein Levels

**Figure 5:**
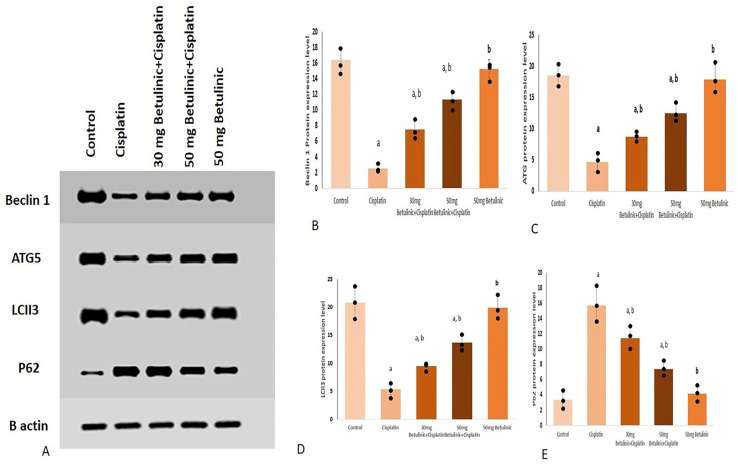
The protein expression level for Beclin 1, ATG5, LCII3 and P62 against B actin (**A**). Individual protein expression levels for (**B**) Beclin 1, (**C**) ATG5, (**D**) LC3II, (**E**) P62. Cisplatin (25 mg/kg, i.p) was used for nephrotoxicity induction. Betulinic acid (30 mg/kg and 50 mg/kg orally) was given for 10 days. Data expressed as mean ± SD (*n* = 3/group), a means significant vs. control, b means significant vs. cisplatin group. *p* ˂ 0.05.

Different protein expression levels were displayed as bands on the gel against the actin bands as shown in Figure 5.

In Figure 5B, the protein expression of Beclin 1 in the cisplatin group was suppressed remarkably (84.7% decrease) compared to the control group. In contrast, betulinic acid dosing (30, 50 mg) exhibited a significant increase in Beclin 1 (3.01 and 4.52-fold increase, respectively) compared with the cisplatin group.

Similarly, cisplatin injection induced a decrease (74.6%) in the ATG5 protein expression compared to the control group. An effect that was partially corrected by the betulinic acid dosing, where it showed a significant increase (1.8 and 2.7-fold increase for 30 and 50 mg betulinic acid, respectively) in ATG5 expression, relative to the cisplatin group values (Figure 5C). Likely, compared with the control group, the cisplatin group manifested a remarkable decrease in LCII3 expression (74.04% decrease). On the other hand, betulinic acid protected the kidney tissue and significantly enhanced the LCII3 protein level (1.75 and 2.54-fold increase for doses 30 and 50 mg, respectively) compared to the cisplatin group (Figure 5D).

Regarding the protein expression level of P62, betulinic acid (30 and 50 mg) proved a significant decrease (27.3% and 52.8% decrease) relative to the cisplatin group, while cisplatin injection significantly increased the P62 levels (Figure 5E).

#### 2.3.5. Histopathological Examination of Kidney Tissues

**Figure 6:**
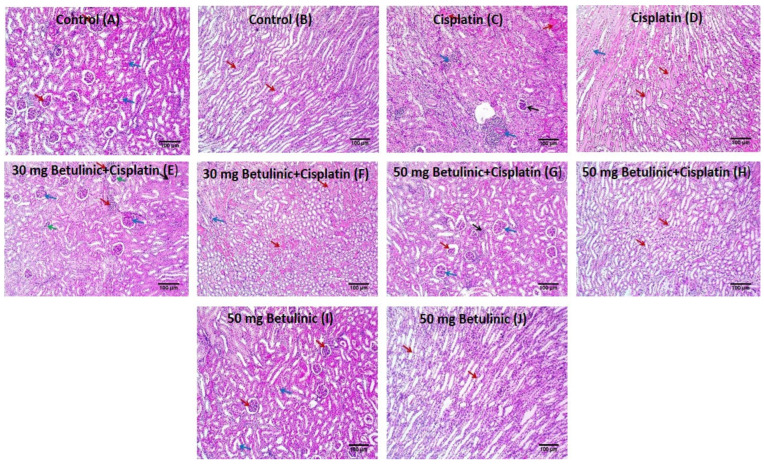
Histopathological findings of the kidney sections [H&E × 100]. (**A**) Cortex of the control group revealed average sized glomeruli (red arrows) with average sized tubules lined surrounding it (blue arrows). (**B**) The medulla section of the control group exhibited columnar cells lining average sized tubules (red arrows). (**C**) Cortex section of the positive control showed destructed glomeruli with one atrophic glomerulus (black arrow) surrounded by high inflammatory cells infiltrate (blue arrows) and some tubules revealing hyaline degeneration (red arrows). (**D**) Medulla section of the positive control exhibited many tubules filled with hyaline casts (red arrows) and inflammatory cells (blue arrow). (**E**) Cortex section of 30 mg/kg Betulinic acid pre-treated group revealed atrophic glomeruli (green arrows), moderate inflammatory cells (red arrows) and some hyalinized tubules (black arrow) and average sized glomeruli (blue arrows). (**F**) Medulla of the 30 mg/kg Betulinic acid pre-treated group revealed tubules filled with hyaline casts (red arrows) and inflammatory cells (blue arrow). (**G**) Cortex of the 50 mg/kg Betulinic acid pre-treated group exhibited few atrophic glomeruli (red arrow), few inflammatory cells (black arrows) and average sized glomeruli (blue arrows). (**H**) Medulla of kidney with 50 mg/kg Betulinic acid pre-treated group revealed average sized tubules lined with columnar cells (red arrows). (**I**) Cortex of Betulinic acid-only pretreated kidney showed average sized tubules lined with columnar cells (blue arrows) surrounding average sized glomeruli (red arrows). (**J**) Medulla of Betulinic acid-only pre-treated kidney revealed average sized tubules lined with columnar cells (red arrows).

As shown in Figure 6, the histopathological examination showed the control tissues with average tubules, while the cisplatin group manifested destructed glomeruli. On the other hand, treatment with two doses of betulinic acid exhibited its cytoprotective action and revealed moderate to few inflammatory cell infiltrations.

#### 2.3.6. Immunohistochemical Evaluation of Interleukin 1 Beta (IL-1B) and Nuclear Factor Kappa B (NFkB) for Different Groups

**Figure 7:**
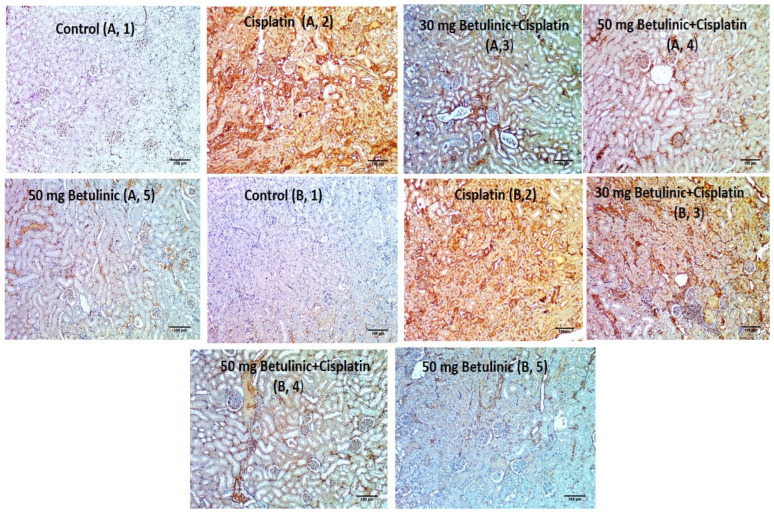
IL-1β (**A**) and NFҡB (**B**) Immunohistochemical findings of the kidney sections. (**A,1**) Section in the kidney of control group showed positive IL-1β expression in less than 5% of cells (score 0) [×100]. (**A,2**) Section in Cisplatin group showed strong IL-1β expression in more than 50% of cells (score 3) [×100]. (**A,3**) Section in 30 mg/kg Betulinic acid pre-treated kidney showed moderate IL-1β expression (score 2) [×100]. (**A,4**) Section in 50 mg/kg Betulinic acid pre-treated showed weak IL-1β expression (score 1) [×100]. (**A,5**) Section in Betulinic acid only treated kidney positive IL-1β expression in less than 5% of cells (score 0) [×100]. (**B,1**) Section in the kidney of control group showed positive NFкB expression in less than 5% of cells (score 0) [×100]. (**B,2**) Section in the Cisplatin group showed strong NFкB expression in more than 50% of cells (score 3) [×100]. (**B,3**) Section in 30 mg/kg Betulinic acid pre-treated kidney showed moderate NFкB expression (score 2) [×100]. (**B,4**) Section 50 mg/kg Betulinic acid pre-treated showed weak NFкB expression (score 1) [×100]. (**B,5**) Section in Betulinic acid only treated kidney positive NFкB expression in less than 5% of cells (score 0) [×100].

Kidney sections from the cisplatin group showed strong expression of both IL-1B and NFкB, while pre-treatment with 30 and 50 mg/kg betulinic acid lessened the score to 2 and 1 (Figure 7).

## 3. Discussion

Unfortunately, there is an increasing number of pathogenic bacteria that acquire antibiotic resistance to many antibiotics all over the world. This is attributed partially to the implausible usage of antimicrobials [14]. Thus, it is urgently needed to elucidate unusual solutions for such problems [15]. Here, we revealed the antibacterial potential of naturally derived material (betulinic acid) toward four bacterial species as a common etiology for UTI. Remarkably, BA exposed antibacterial action with MIC values of 128 and 512 µg/mL. The possible effect of BA on the membrane integrity of the tested bacterial isolates was investigated.

Certain antibacterial substances, like the bioactive compounds of certain plant extracts, affect the integrity of the bacterial membranes and lead to the release of cytoplasmic substances from the interior of the cells [16,17]. This, in turn, affects the viability of the bacterial cells. Therefore, we investigated betulinic acid’s effect on the tested bacteria’s bacterial membrane integrity. It was found to adversely affect the integrity of the studied isolate membranes.

Nephrotoxicity is defined as the decline of normal kidney function. It is associated with AKI, which is commonly caused by the administration of nephrotoxic drugs like cisplatin [18]. The current study is the first of its kind that aimed to investigate the reno-protective potentiality of BA and the possible underlying mechanisms that manage such an effect.

The renal function deterioration is manifested through the marked increase in the serum blood urea nitrogen, creatinine, and NGAL levels [19,20]. It has been speculated that mice treated with cisplatin showed a remarkable increase in the indicators of renal function loss, represented in serum NGAL, creatinine, and KIM1 [21].

In the present study, cisplatin clearly caused profound nephrotoxicity, reflected by the remarkable rise in serum creatinine, NGAL, and KIM1 values and strong histopathological manifestations, including damaged glomeruli and inflammatory cell infiltration. Pretreatment with BA pronouncedly decreases the renal toxicity-associated biomarkers and efficiently lessens histopathological inflammation.

Earlier reports speculated that oxidative stress is one of the tools through which cisplatin induces kidney injury and cell death. It was proven that cisplatin increases kidney MDA levels and decreases the antioxidant SOD activity level [22]. The present study was in alignment, as the cisplatin-treated group showed an increase in MDA and a decrease in renal SOD levels. Whereas the BA-treated groups showed a profound decrease in oxidative stress with SOD activity leveling up.

As a result of oxidative stress activation, NFкB is released and binds to DNA in the nuclei to increase the transcription of different inflammatory genes, including cytokine [2]. In the present study, upon injecting cisplatin, NFкB increased, as shown by the immunohistochemical illustration, where strong expression was stated with a score of three, supporting previous studies [23,24].

The current findings illustrated that cisplatin provoked IL-1 β expression manifested as strong immunohistochemical staining of IL-1β. However, BA counteracted the cisplatin action and lessened the staining intensity. This effect could be explained by the ability of NFкB to trigger the activation of IL-1β via different pathways, including inhibition of inflammasomes-mediated Il-1β activation in kidney tissues with acute injury [25]. IL-1β is a main inflammatory cytokine that plays a crucial role in cell death activation and production of other inflammatory mediators.

Autophagy is induced in response to cellular stress when the kidney is diseased or exposed to insults or toxins, such as cisplatin. Autophagy has been reported to get activated after a strong signal of oxidative stress and is generally considered pivotal in promoting cell survival and protecting against acute cisplatin nephrotoxicity [26].

Autophagy is mainly regulated by autophagy-specific genes (ATGs) that include Beclin 1. During autophagy initiation and nucleation, Beclin 1 is recruited and activated. Then, the formation and elongation of autophagosomes are accompanied by the ability of Beclin 1 to activate the membrane bound LC3II active form. Meanwhile, ATG5 is recruited and helps activate the closure of the autophagosomes [27]. It was also stated that the cisplatin injection was associated with suppressing autophagic LC3II expression [28], and the more autophagy got inhibited, the worse kidney function got [26,29,30]. In the current study, mice treated with cisplatin showed substantial repression in the gene expression of Beclin 1, ATG5, as well as inhibition of LC3-II gene expression. This effect was in line with previous reports [31].

The adaptor protein p62 is a well-known facilitator of the autophagic degradation of ubiquitinated protein aggregates in lysosomes. It also facilitates the activation of caspases and hence apoptosis. Interestingly, activated autophagy, through negative feedback, leads to decrease p62 level and defective or impaired autophagy is associated with accumulation of p62 [32]. Herein, the cisplatin-treated group exhibited a significant increase in p62 along with inhibition of the autophagy process, which was in alignment with the earlier report [28]. In contrast, pretreatment with BA exhibited a marked management of autophagy, shown as a significantly increased gene expression of Beclin 1, ATG5, LC3-II and a significant decrease in p62 gene expression.

As mentioned by Gong et al. 2020 [33], autophagy is not only activated by oxidative stress but can also, accordingly, halt the oxidative stress process and NFкB activation. This was confirmed in the current findings as the increase in autophagy biomarkers was associated with a decrease in NFкB staining and oxidative stress signal. Thus, our findings were in line and suggested a possible interplay between autophagy and oxidative stress-related mediators.

## 4. Materials and Methods

### 4.1. Plant and Chemicals

The total aerial parts of the *Silene succulent* Forssk. plant were gathered on the northwest coast of Matrouh in April 2020. *Silene succulent* was kindly established by Dr. Esraa Ammar, Department of Plant Ecology, Faculty of Science, Tanta University. A voucher specimen (PG-A-SS-01) was deposited in the herbarium of Tanta University. Mylan Pharmaceuticals Co. was the manufacturer of the cisplatin injection. All chemicals were obtained from Merck (Rahway, NJ, USA).

### 4.2. Extraction and Isolation of BA

The aerial parts were dried and ground then 450 g of powder was macerated with 70% methanol (3 × 3 L). The methanolic extract was concentrated under a vacuum to yield the residue. The concentrated residue was then suspended in distilled water and partitioned with pet-ether, ethyl acetate, and n-butanol. Ten grams of ethyl acetate fraction was subjected to (VLC) Silica gel column using gradient elution and *n*-hexane: ethyl acetate as mobile phase affording six subfractions (Sa–Sf). The fifth subfraction (Se-1.4 g, eluted at 50%) was subjected to further purification using Sephadex LH-20 (1:1-DCM: MeOH) several times to obtain betulinic acid powder for the first time from this plant.

### 4.3. Antibacterial Susceptibility

Agar well diffusion was utilized [34] to illuminate the antibacterial action of betulinic acid on the uropathogenic bacteria: *Pseudomonas aeruginosa* (ATCC 27853), *Escherichia coli* (ATCC 25922), *Proteus mirabilis* (ATCC 35659), and *Klebsiella pneumoniae* (ATCC 13883) isolates. After punching three wells in the Muller-Hinton agar, they were filled with betulinic acid, ciprofloxacin, and dimethyl sulfoxide (10%). The MICs of betulinic acid were detected using broth microdilution technique using Muller-Hinton broth (Oxoid, UK) in microtitration plates. The lowest concentration of betulinic acid, which caused an inhibition of bacterial growth, was documented as MIC value [35].

### 4.4. Membrane Integrity Test

The influence of betulinic acid on the bacterial membrane integrity was investigated by detecting the release of the materials that have absorbance at 260 nm according to the method previously described [8]. Briefly, the OD of the bacterial suspensions was adjusted to 0.4. Then, they were centrifuged, and the obtained pellets were suspended in a sodium chloride solution (0.5%). A UV/V spectrophotometer was used for monitoring the absorbance at 260 nm throughout time.

### 4.5. Animal Handling

Male albino mice (*n* = 30, weight 21–25 g) were purchased from the animal house of the faculty of veterinary medicine (Cairo, Egypt). The mice were kept for one week for acclimatization. The mice were fed ad libitum with regular food and water. Animal handling followed the Guidelines for the Management and Use of Laboratory Animals (Approval code TP/RE/2/23P-008).

The mice were designated into five groups (6 per group) and treated for ten days. Group I served as the normal control group. Group II was treated with cisplatin (25 mg/kg, single I.P injection) on the seventh day [36] and served as the positive control. Group III was treated with cisplatin (a single I.P injection) on the seventh day and treated orally with 30 mg/kg betulinic acid for a week before and 72 h following the cisplatin injection. Group IV was treated with a single I.P injection of cisplatin on the seventh day and treated orally with 50 mg/kg betulinic acid for seven days before and three days after the cisplatin injection. Group V was treated with 50 mg of betulinic acid only for ten days [37,38].

### 4.6. Sample Collection

Mice were anesthetized with Diethyl ether three days following the cisplatin treatment. Carefully, we collected blood samples, from which serum was obtained and kept at 20 °C for further biochemical assessments. A small portion of the kidneys was kept in 10% formalin for further histological and immunohistochemical investigations. For the biochemical investigation of the tissue content, the remaining portions of the kidneys were kept frozen in liquid nitrogen.

### 4.7. Determination of KIM-1, Serum Creatinine, and NGAL

Legend MAX Mouse ELISA Kit for KIM-1 and NGAL obtained from (ab119596, Abcam, Cambridge, UK) and (ab119601, Abcam, Cambridge, UK), respectively, were used. The manufacturer protocol was followed to measure both protein levels. A colorimetric kit was obtained to measure serum creatinine (700460, Cayman, Ann Arbor, MI, USA) according to the attached protocol.

### 4.8. Lipid Peroxidation

The renal tissue homogenate content of MDA was evaluated using kits obtained from Biodiagnostic, Giza, Egypt. With cat number MD 25 29.

### 4.9. Superoxide Dismutase Activity

The renal homogenate was used to determine the SOD activity using a commercially available kit (SD 25 21, Biodiagnostic, Egypt).

### 4.10. qRT-PCR for Beclin-1, P62, LC3II, ATG5 Genes

According to the manufacturer’s protocol, gene expression of the Beclin-1, P62, LC3II, and ATG5 genes was measured. Steps and primer sequences were listed in the Supplementary Materials (Table S1).

The thermal cycling program was set according to the previously reported [39]. The 2^−ΔΔCT^ method was used to obtain the relative mRNA fold expression using GAPDH for normalization [40].

### 4.11. Western Blotting

After extracting the protein using a TriFast kit (Peqlab, VWR Company, Radnor, PA, USA), the extracted protein samples were loaded on sodium dodecyl sulfate-polyacrylamide gel electrophoresis (SDS-PAGE). For blotting, electrophoresed proteins on SDS-PAGE were transferred to a Hybond ™ nylon membrane (GE Healthcare, Chicago, IL, USA) via TE62 Standard Transfer Tank with Cooling Chamber (Hoefer Inc. and incubated for 1 h at room temperature in Blocking Solution. Additionally, β-actin (abcam, ab8227) was applied as a housekeeping protein. Then, the membrane was incubated overnight at 4 °C in Antibody Solution containing Anti-LC3II (ab192890, abcam), Anti-P62 (ab109012, abcam), NTI-ATG5, (ab307843, abcam), anti-Beclin 1 (ab223348, abcam) antibody, 15 KDa followed by washing the membrane with Blotting Buffer. Finally, the membrane was incubated for 1 h at room temperature in an Antibody Solution containing appropriate dilution of HRP-conjugated secondary antibody (Antibody concentration. 0.1–0.5 microgram/mL) [41]. The gel documentation system (Geldoc-it, UVP, Bonn, Germany) was applied for data analysis using Totallab analysis software, ww.totallab.com, (accessed on 30 December 2022) (Ver.1.0.1).

### 4.12. Histopathological Study

Kidney sections were viewed under a light microscope after staining, according to the earlier stated technique [42].

### 4.13. Immunohistochemical Evaluation

For immunohistochemical staining, the NFкB and IL-1β expressions were determined in the kidney tissues using ABclonal Technology kits (Woburn, MA, USA). Based on the positive staining percentages, the applied scores were as follows: 0 denotes the lack of immune-positive cells, and 1 denotes the presence of up to 25% of immune-positive cells. Score 2 denotes the presence of cells that have an immune stain of 11–50%, score 3 the presence of cells that have an immunological stain of 51–75%, and score 4 the presence of cells that have an immune stain of 76–100% [43].

### 4.14. Statistical Analysis

The data was provided as a mean ± standard deviation. A one-way analysis of variance (ANOVA) was utilized to compare different groups, followed by an LSD post hoc test. *p* < 0.05 was used as the significant level. The statistical analysis was carried out using SPSS version 25.

## 5. Conclusions

To the best of our knowledge, this study was the first to shed light on the impact of BA on cisplatin-induced acute kidney injury focusing on pyroptosis, oxidative stress and accompanied inflammation. In a nutshell, BA exhibited a promising reno-protective effect on cisplatin-induced nephrotoxicity in mice, as manifested by improved histopathological changes related to cisplatin nephrotoxicity as well as a substantial decrease in serum creatinine, KIM-1, and NGAL tissue levels. An effect that could be explained by suppressing oxidative stress and inflammation along with modulating the autophagy pathway. BA revealed antibacterial action toward uropathogenic bacteria by affecting the membrane integrity of the tested bacteria.

## Figures and Tables

**Table 1:** ^1^H, ^13^C and DEPT135 spectral data (Pyridine-*d_5_*) (400 MHz for ^1^H and 100 MHz for ^13^C).

Position	^13^C	^1^H (*J* in Hz)
1	39.67 (CH_2_)	1.69 (2H, m)
2	28.66 (CH_2_)	1.96 (2H, m)
3	78.50 (CH)	3.47 (1H, m)
4	39.89 (C)	-
5	56.31 (CH_2_)	-
6	19.16 (CH_2_)	-
7	35.23 (CH_2_)	2.26 (1H, brs)
8	41.50 (C)	-
9	51.35 (CH)	-
10	37.90 (C)	-
11	21.60 (CH_2_)	-
12	26.50 (CH_2_)	-
13	38.99 (CH)	-
14	43.23 (C)	-
15	31.61 (CH_2_)	2.62 (2H, m)
16	33.27 (CH_2_)	2.65 (2H, m)
17	57.01 (C)	-
18	48.14 (CH)	3.54 (1H, m)
19	50.15 (CH)	1.77(1H, m)
20	151.67 (C)	-
21	30.65 (CH_2_)	-
22	37.94 (CH_2_)	2.77 (2H, m)
23	16.80 (CH_3_)	1.23 (3H, s)
24	29.04 (CH_3_)	1.23 (3H, s)
25	16.80 (CH_3_)	1.85 (3H, s)
26	16.71 (CH_3_)	1.02 (3H, s)
27	15.29 (CH_3_)	0.84 (3H, s)
28	179.22 (C)	-
29	110.31 (CH_2_)	4.96 (1H, brs) 4.78 (1H, brs)
30	19.87 (CH_3_)	1.81 (3H, s)

**Table 2:** The MIC values of betulinic acid on the uropathogenic bacterial isolates.

Bacterial Species	*Pseudomonas aeruginosa*	*Escherichia coli*	*Proteus mirabilis*	*Klebsiella pneumoniae*
MIC values (µg/mL)	256	512	128	256

**Table 3:** Effect of Betulinic acid pretreatment on cisplatin-induced nephrotoxicity.

	Kidney MDA (nm/g Tissue)	Kidney SOD Activity (U/mg Tissue)
Control	137.86 ± 8.9	3.04 ± 0.44
Cisplatin	265.58 ± 15.75 ^a^	1.47 ± 0.18 ^a^
30 mg Betulinic + Cisplatin	209.56 ± 9.18 ^a,b^	1.98 ± 0.17 ^a,b^
50 mg Betulinic + Cisplatin	173.66 ± 10.13 ^a,b^	2.4 ± 0.12 ^a,b^
50 mg Betulinic only	135.9 ± 9 ^b^	3.12 ± 0.61 ^b^

Cisplatin (25 mg/kg, i.p) was used for nephrotoxicity induction. Betulinic acid (30 mg/kg and 50 mg/kg, orally) was given for 10 days. Data expressed as mean ± SD (*n* = 6/group), ^a^: means significant vs. control, ^b^: means significant vs. cisplatin group. *p* ˂ 0.05.

## Data Availability

All data is contained within the article and Supplementary Material.

## Supplementary Materials

The following supporting information can be downloaded at: https://www.mdpi.com/article/10.3390/ph16081180/s1; Table S1: Beclin 1, p62, ATG5, LC3II genes primer sequences. Figure S1: The HR-ESI-MS of BA. Refs. [40,44,45,46,47] are in the Supplementary Materials part.
