# Supplementary material for: Protective Role of Betulinic Acid against Cisplatin-Induced Nephrotoxicity and Its Antibacterial Potential toward Uropathogenic Bacteria
Source: Pharmaceuticals (Basel). 2023 Aug 18;16(8):1180. doi: 10.3390/ph16081180 (PMC10458273; doi:10.3390/ph16081180)
Supplement: Supplementary file 1 [file pharmaceuticals-16-01180-s001.zip › pharmaceuticals-2466040-supplementary.pdf]

Methodology

The total RNA from kidney samples was purified using TRIzols Reagent (Life Technologies, USA). In a two-step RT-PCR experiment, 1µg of total RNA was reverse-transcribed into single-stranded complementary DNA using the QuantiTects Reverse Transcription Kit (Qiagen, USA) and a random primer hexamer. Maximas SYBR Green/Fluorescein qPCR Master Mix was used to amplify C-DNA amplicons through specific primers.

Table S1. Beclin1, p62, ATG5, LC3II genes primer sequences.

Gene	primer sequences	References
Beclin-1	5′-ATACTGTTCTGGGGGTTTGCG-3′<br>5′-GTCTCTCCTTTTTCCACCTCTTC-3′.	(Liu, Huang, Liu, Song, & Xiao, 2020)
P62	5′- AGG GAA CAC AGC AAG CT -3′<br>5′- GCC AAA GTG TCC ATG TTT CA -3′	(Crippa et al., 2013)
GAPDH	5′-TGACAACTTTGGTATCGTGGAAGG-3′;<br>5′-AGGCAGGGATGATGTTCTGGAGAG-3′.	(Yang, Yu, Liu, Yang, & Tao, 2020)
LC3II	5′-AACATGAGCGAGTTGGTCAAG-3′;<br>5′-GCTCGTAGATGTCCGCGAT-3′;	
ATG5	5′CCAAGCTTCTAATACGACTCACTATAGG<br>GAGAATGACAGATGACAAAGATGTGC-3′<br>5′-TCAATCTGTTGGCTGGGGGACA-3′	(Kim et al., 2014)

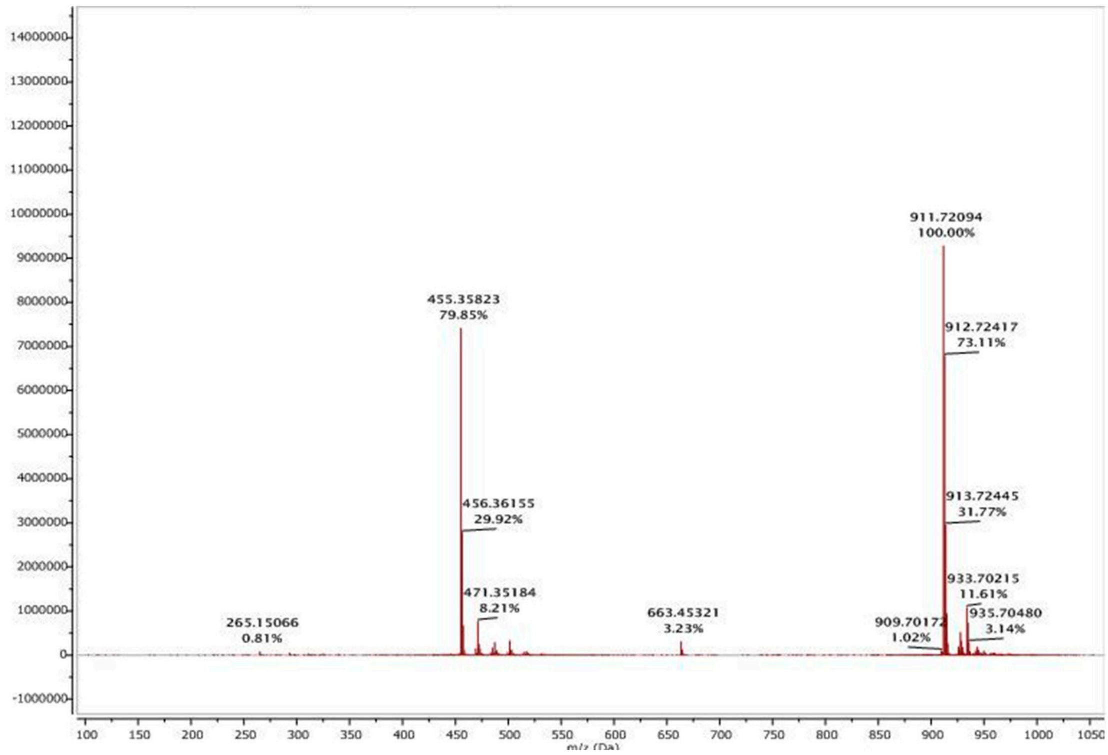

Figure S1. The HR-ESI-MS of BA.

References

Crippa, V., Boncoraglio, A., Galbiati, M., Aggarwal, T., Rusmini, P., Giorgetti, E., . . . Poletti, A. (2013). Differential autophagy power in the spinal cord and muscle of transgenic ALS mice. *Frontiers in cellular neuroscience, 7*, 234.

Kim, C., Kim, W., Lee, H., Ji, E., Choe, Y.-J., Martindale, J. L., . . . Nam, S. W. (2014). The RNA-binding protein HuD regulates autophagosome formation in pancreatic β cells by promoting autophagy-related gene 5 expression. *Journal of Biological Chemistry, 289*(1), 112-121.

Liu, S., Huang, X., Liu, Y., Song, D., & Xiao, Y. (2020). Functional analysis of miRNAs combined with TGF-β1/Smad3 inhibitor in an intrauterine rat adhesion cell model. *Molecular and Cellular Biochemistry, 470*, 15-28.

Livak, K. J., & Schmittgen, T. D. (2001). Analysis of relative gene expression data using real-time quantitative PCR and the 2− ΔΔCT method. *methods, 25*(4), 402-408.

Yang, Z., Yu, W., Liu, B., Yang, M., & Tao, H. (2020). Estrogen receptor β induces autophagy of osteosarcoma through the mTOR signaling pathway. *Journal of Orthopaedic Surgery and Research, 15*, 1-7.
